# Prevalence of pathogenic variants in cancer‐predisposing genes in second cancer after childhood solid cancers

**DOI:** 10.1002/cam4.5835

**Published:** 2023-04-06

**Authors:** Masanori Yoshida, Kazuhiko Nakabayashi, Wentao Yang, Aiko Sato‐Otsubo, Shin‐ichi Tsujimoto, Hiroko Ogata‐Kawata, Tomoko Kawai, Keisuke Ishiwata, Mika Sakamoto, Kohji Okamura, Kaoru Yoshida, Ryota Shirai, Tomoo Osumi, Chikako Kiyotani, Yoko Shioda, Keita Terashima, Sae Ishimaru, Yuki Yuza, Masatoshi Takagi, Yuki Arakawa, Toshihiko Imamura, Daisuke Hasegawa, Akiko Inoue, Takako Yoshioka, Shuichi Ito, Daisuke Tomizawa, Katsuyoshi Koh, Kimikazu Matsumoto, Nobutaka Kiyokawa, Seishi Ogawa, Atsushi Manabe, Akira Niwa, Kenichiro Hata, Jun J. Yang, Motohiro Kato

**Affiliations:** ^1^ Department of Pediatric Hematology and Oncology Research Research Institute, National Center for Child Health and Development Tokyo Japan; ^2^ Department of Pediatrics, Graduate School of Medicine Yokohama City University Yokohama Japan; ^3^ Department of Maternal‐Fetal Biology Research Institute, National Center for Child Health and Development Tokyo Japan; ^4^ Department of Pharmacy and Pharmaceutical Sciences St. Jude Children's Research Hospital Tennessee Memphis USA; ^5^ Department of Pediatrics, Graduate School of Medicine The University of Tokyo Tokyo Japan; ^6^ Medical Genome Center Research Institute, National Center for Child Health and Development Tokyo Japan; ^7^ Department of Systems BioMedicine Research Institute, National Center for Child Health and Development Tokyo Japan; ^8^ Children's Cancer Center National Center for Child Health and Development Tokyo Japan; ^9^ Department of Hematology/Oncology Tokyo Metropolitan Children's Medical Center Tokyo Japan; ^10^ Trial and Data Center Princess Máxima Center for Pediatric Oncology Utrecht the Netherlands; ^11^ Department of Pediatrics and Developmental Biology Graduate School of Medical and Dental Sciences, Tokyo Medical and Dental University (TMDU) Tokyo Japan; ^12^ Department of Hematology/Oncology Saitama Children's Medical Center Saitama Japan; ^13^ Department of Pediatrics Kyoto Prefectural University of Medicine, Graduate School of Medical Science Kyoto Japan; ^14^ Department of Pediatrics St. Luke's International Hospital Tokyo Japan; ^15^ Department of Pediatrics Osaka Medical and Pharmaceutical University Takatsuki Japan; ^16^ Department of Pathology National Center for Child Health and Development Tokyo Japan; ^17^ Department of Pathology and Tumor Biology Graduate School of Medicine, Kyoto University Kyoto Japan; ^18^ Department of Pediatrics Hokkaido University Graduate School of Medicine Sapporo Japan; ^19^ Department of Clinical Application, Center for iPS Cell Research and Application Kyoto University Kyoto Japan; ^20^ Department of Human Molecular Genetics, Gunma University Graduate School of Medicine Maebashi Japan; ^21^ Department of Oncology St. Jude Children's Research Hospital Memphis USA; ^22^ Department of Pediatrics The University of Tokyo Tokyo Japan

**Keywords:** cancer predisposition, childhood solid cancer, second malignant neoplasms

## Abstract

**Background:**

Second malignant neoplasms (SMNs) are one of the most severe late complications after pediatric cancer treatment. However, the effect of genetic variation on SMNs remains unclear. In this study, we revealed germline genetic factors that contribute to the development of SMNs after treatment of pediatric solid tumors.

**Methods:**

We performed whole‐exome sequencing in 14 pediatric patients with SMNs, including three brain tumors.

**Results:**

Our analysis revealed that five of 14 (35.7%) patients had pathogenic germline variants in cancer‐predisposing genes (CPGs), which was significantly higher than in the control cohort (*p* < 0.01). The identified genes with variants were *TP53* (*n* = 2), *DICER1* (*n* = 1), *PMS2* (*n* = 1), and *PTCH1* (*n* = 1). In terms of the type of subsequent cancer, leukemia and multiple episodes of SMN had an exceptionally high rate of CPG pathogenic variants. None of the patients with germline variants had a family history of SMN development. Mutational signature analysis showed that platinum drugs contributed to the development of SMN in three cases, which suggests the role of platinum agents in SMN development.

**Conclusions:**

We highlight that overlapping effects of genetic background and primary cancer treatment contribute to the development of second cancers after treatment of pediatric solid tumors. A comprehensive analysis of germline and tumor samples may be useful to predict the risk of secondary cancers.

## Graphical abstract

Undiagnosed cancer predisposition syndromes are frequently present in patients who develop second cancer after treatment for childhood solid tumors. Overlapping effects of genetic background and primary cancer treatment contribute to the development of second cancers.

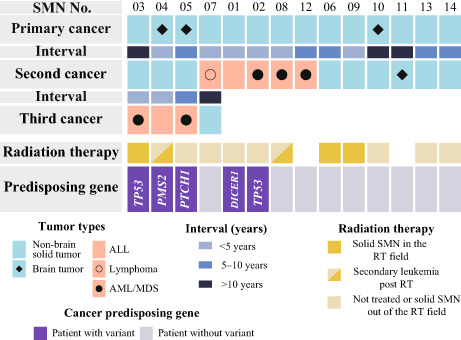

## 1 INTRODUCTION

Second malignant neoplasms (SMNs) are the most severe late complications in childhood cancer survivors and are the leading cause of death in long‐term survivors.^1^ The cumulative incidence of SMNs does not reach plateau even at 10 years after the treatment of primary cancer, more than 10% of long‐term survivors develop SMNs.^1^, ^2^, ^3^

Several known risk factors for SMN development have been reported. In addition to age at the first cancer, gender, radiation therapy, and DNA‐damaging agents (most notably alkylating agents) are established risk factors for SMNs.^4^, ^5^, ^6^, ^7^ In addition to these factors, germline pathogenic variants of cancer‐related genes are associated with the development of SMNs. Recent comprehensive analyses have revealed that pediatric cancer patients have a high frequency of germline pathogenic variants in cancer‐predisposing genes (CPGs).^8^, ^9^ Since a history of cancer is often observed in the family members of SMNs,^10^ genetic variations in CPGs are believed to be associated with the development of SMNs after childhood cancer. For example, *TP53* variants are frequently found in the patient with pediatric SMNs after treatment for solid tumors and acute lymphoblastic leukemia (ALL).^11^, ^12^ Additionally, several studies have reported the results of germline variant analyses in the development of SMNs. A comprehensive study of childhood cancer survivors reported that 6.4% of the patients had germline pathogenic variants of CPGs.^13^ In a study of pediatric therapy‐related myeloid neoplasms, pathogenic or likely pathogenic variants were identified in 13 of 84 patients (15%).^14^

Thus, the influence of germline sequence alterations in the development of SMNs is gradually being elucidated, but the data are still limited. Here, we report an analysis of germline alterations in CPGs and somatic mutation profiles in pediatric patients with SMNs after treatment for solid tumors.

## 2 PATIENTS AND METHODS

### 2.1 Patient enrollment

Patients who met the following eligibility criteria were enrolled in this retrospective study: (i) developed SMNs after treatment for pediatric solid tumors, (ii) specimens were available, and (iii) provided required informed consent. SMN is defined as a new, independent cancer that occurs in a person who has had cancer in the past. Germline samples (*n* = 14) with available somatic samples (*n* = 5) that met these criteria were obtained.

### 2.2 Germline analysis of pathogenic variants of CPGs

This retrospective study was designed to clarify the genetic risk factors for SMNs. To investigate potential pathogenic variants of CPGs in SMN cases, we performed whole‐exome sequencing (WES) in 14 germline samples collected from the tumor‐free specimens (Supplementary method and Table S1). Complete methods of sample preparation and WES are shown in detail in the Supplementary files. The selection of the CPG genes and the evaluation of the pathogenicity were performed as previously reported.^15^ In brief, variants of 162 known CPGs (Table S2) listed in previous reports^9^, ^16^ were extracted from each sample. Then, the pathogenicity of each variant was manually evaluated using online databases and the guideline of the American College of Medical Genetics and Genomics^17^ (detailed in the Supplementary methods). Detailed genomic data are available on request for corresponding author.

### 2.3 Comparison of the prevalence of germline variants between the experimental group and control samples

To compare the prevalence of germline variants between the experimental group and the control cohort, WES data of 104 adults with no cancer history or a family history of hematologic disorders, consecutively enrolled in the National Center Biobank Network (NCBN) project were analyzed using the same process as that used for the experimental cohort.^15^

### 2.4 Genomic analysis of SMN samples

To identify somatic mutations in SMN specimens, available tumor samples at SMN diagnosis from five patients were analyzed using WES. Using paired tumor–normal WES data, somatic mutations were extracted. Driver mutations were identified by reference to the Catalogue of Somatic Mutations in Cancer (COSMIC, http://cancer.sanger.ac.uk/cancergenome/projects/cosmic), NCBI ClinVar (http://www.ncbi.nlm.nih.gov/clinvar/), and previous reports.

### 2.5 Copy number analysis

We also analyzed the WES data using CNVkit (version 0.9.6) to estimate the copy number status.^18^ In this analysis, the number of reads mapped to each target region of the tumor genome was calculated and compared with the value obtained from multiple normal reference samples. Copy number segmentation was performed using the circular binary segmentation method with default parameter settings.

### 2.6 Tumor mutational burden analysis

Tumor mutational burden (TMB) was calculated as the total number of somatic, coding, base substitution, and indel mutations per mega base, using paired tumor–normal WES data.

### 2.7 Mutational signature analysis

To investigate the etiology of SMN development, mutational signature analysis was performed using a previously described method.^15^ Known mutational signatures were obtained from the COSMIC database (Mutational signatures V3, synapse.org ID: syn12009743) and from Li et al.^19^

### 2.8 Statistical analysis

The comparison of the prevalence of pathogenic variants between SMN patients and NCBN controls was performed using Fisher's exact test. A *p* value of <0.05 was considered statistically significant.

## 3 RESULTS

### 3.1 Patient characteristics

**FIGURE 1:**
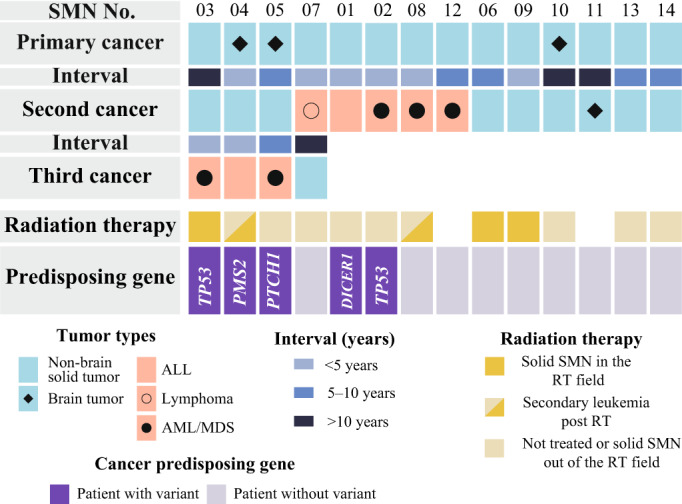
Overview of patient characteristics and genetic variation in subsequent malignant neoplasms. The subtype of primary and subsequent malignant neoplasms (SMN), the interval of primary cancer and SMN, and cancer‐predisposing genes variant are shown.

The characteristics of the 14 patients with SMNs are presented in Figure 1 and in Tables 1 and 2. Brain tumors (*n* = 3) and nonbrain solid tumors (*n* = 11) were the first cancers in these patients. As for subsequent cancers, five patients had leukemia (*n* = 4) or lymphoma (*n* = 1), and nine patients had solid tumors, including a brain tumor (*n* = 1) and nonbrain solid tumors (*n* = 8). Four of the 14 patients had multiple episodes of SMNs including both solid and hematologic malignancies (SMN03–05, and 07). The median age at diagnosis of primary cancer and SMNs was 3 years (range, 9 months–13 years) and 10 years (range, 4–19 years), respectively. The median time from diagnosis of the primary malignancy to diagnosis of SMN was 6 years (range, 1–11 years). Radiation therapy was administered in five cases. Three patients developed a second solid tumor within the radiation fields, while the remaining SMNs were hematological (Figure 1; Table 2).

**TABLE 1:** Clinical characteristics of the SMN patient cohort.

Characteristics			
Total, *n*	14		
Primary cancer			
Median age at diagnosis, years (range)	3 (0–13)		
CNS tumor	3		
Non‐CNS tumor	11		
SMN			
Median age at diagnosis, years (range)	10 (4–19)		
Median interval from primary cancer, years (range)	5.5 (1–11)		
Hematologic malignancies, n			
ALL	1		
AML/MDS	3		
Lymphoma	1		
Solid tumor, *n*			
CNS tumor	1		
Non‐CNS tumor	8		
Third malignancy, *n*	4		
Median age at diagnosis, years (range)	14 (12–21)		
Median interval from second cancer, years (range)	4 (2–11)		
Hematologic malignancies, *n*	4		
ALL	1		
AML/MDS	2		
Solid tumor, *n*	1		
Non‐CNS tumor	1		

Abbreviations: ALL, acute lymphoblastic leukemia; AML, acute myeloid leukemia; CNS, central nervous system; MDS, myelodysplastic syndrome; SMN, second malignant neoplasms.

**TABLE 2:** Patient profiles.

SMN No.	Primary cancer subtypes	Age at primary cancer^a^	Irradiation for primary cancer	SMNs in irradiation field	Second cancer subtypes	Age at second cancer^a^	Interval^b^	Third cancer	Outcome
01	PPB	2	Nonirradiated	No	BCP‐ALL	4	2		Dead
02	ACC	3	Nonirradiated	No	AML	5	2		Dead
03	RMS	2	Irradiated	Yes/NA^c^	OS	13	11	AML	Dead
04	MB	9	Irradiated	No/NA^c^	DC	10	1	BCP‐ALL	Alive
05	MB	1	Nonirradiated	No	OS	7	6	AML	Alive
06	NB	1	Irradiated	Yes	Thyroid cancer	10	9		Alive
07	RMS	6	Irradiated	No	T‐LBL	10	4	RCC	Alive
08	RMS	13	Irradiated	NA	AML	16	3		Dead
09	WT	4	Irradiated	Yes	Thyroid cancer	8	4		Alive
10	Ependymoma	2	Irradiated	No	RCC	13	11		Alive
11	RMS	9	NA	NA	AA	19	10		Alive
12	ES	9	NA	NA	AML	14	5		Alive
13	ES	3	Nonirradiated	No	Bladder cancer	9	6		Alive
14	NB	9 months	Nonirradiated	No	SPN	8	7		Alive

Abbreviations: AA, anaplastic astrocytoma; ACC, adrenal cortex cancer; AML, acute myeloid leukemia; BCP‐ALL, B‐cell precursor acute lymphoblastic leukemia; DC, duodenal cancer; ES, Ewing sarcoma; MB, medulloblastoma; NB, neuroblastoma; OS, osteosarcoma; PPB, pleuropulmonary blastoma; RCC, renal cell carcinoma; RMS, rhabdomyosarcoma; SPN, solid pseudopapillary neoplasm; T‐LBL, T‐lymphoblastic lymphoma; WT, Wilms tumor.

^a^ Years.

^b^ Duration from primary cancer to SMN (years).

^c^ Since the third cancer was leukemia, determination of the irradiation range was not applicable.

### 3.2 Germline pathogenic variants in CPGs

WES identified 25 nonsilent germline variants in the 110 cancer‐associated genes with a dominant inheritance pattern. Five variants were deemed to be pathogenic and were detected in five patients (Figure 1; Table S3). Notably, neither of the five patients with pathogenic variants had a family history of cancer at the time of SMN development. Pathogenic variants were detected in *TP53* (*n* = 2), *DICER1* (*n* = 1), *PMS2* (*n* = 1), and *PTCH1* (*n* = 1). Four patients experienced a third SMN, and of these, three had pathogenic variants (SMN 04–06). In terms of treatment for their prior malignancy, three of five patients with a CPG variant had not received radiation therapy (Figure 1).

**FIGURE 2:**
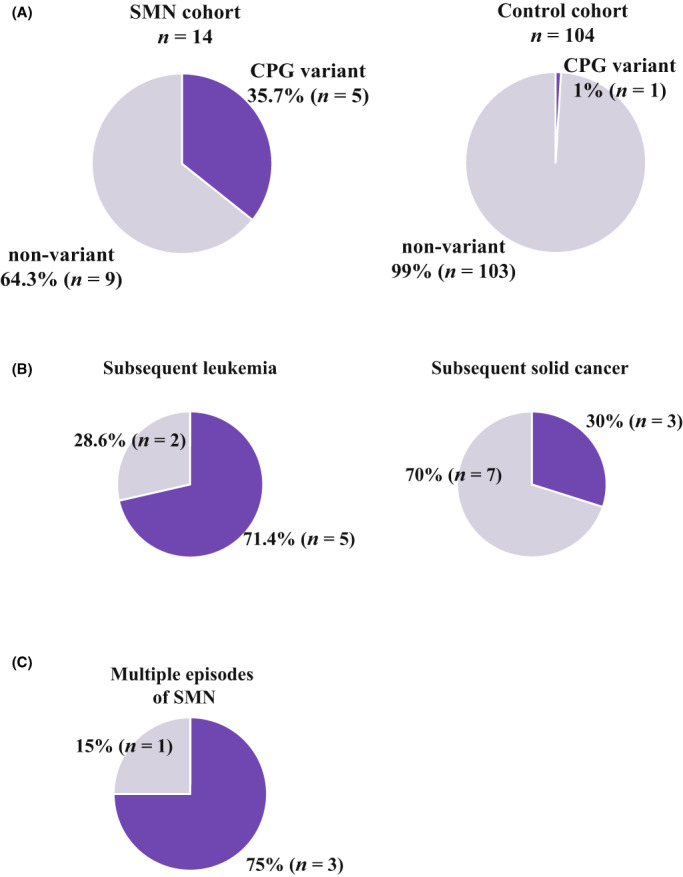
Frequency of germline pathogenic variants of CPGs in pediatric patients with SMNs. CPG, cancer predisposing gene; SMN, second/subsequent malignant neoplasm. CPG variant and nonvariant are shown with dark and light purple colors, respectively. (A) The comparison of the frequency of CPG pathogenic variant between second cancer (SMN) cohort and NCBN noncancer control cohort. (B) CPG variant frequencies by cancer type of SMNs. Brain and nonbrain tumor were categorized as solid cancers. Leukemia and lymphoma were categorized as hematological malignancies. Patients with multiple SMN episodes, including both hematological and solid SMNs, were counted in both hematological and solid SMN in the subsequent cancer categories. C, CPG variant frequency in patients with multiple episodes of SMN.

The prevalence of pathogenic variants in the SMN cohort was estimated to be 35.7% (5 of 14), which was significantly higher than that in the 104 control cases in the NCBN cohort (1.0%; *p* < 0.01) (Figure 2A; Table S4). In terms of cancer type, subsequent leukemia cases and cases with multiple episodes of SMN exhibited a particularly high rate of CPG pathogenic variants (71.4% and 75%) (Figure 2B,C).

Of the 52 genes with a recessive inheritance pattern, none were detected as homozygous or compound heterozygous mutations in either the SMN or the control cohort.

### 3.3 Genomic features of SMN tumor samples

**FIGURE 3:**
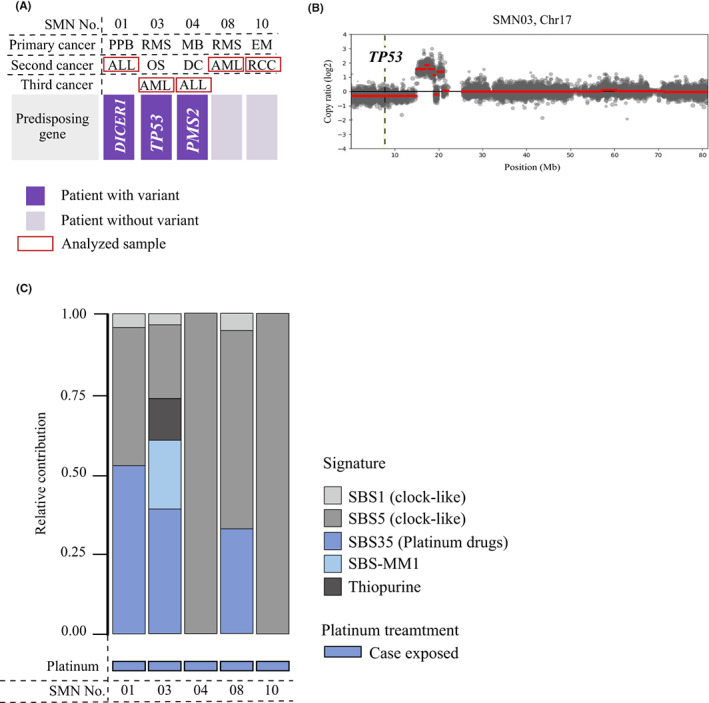
Genetic analysis of SMN tumor samples relative contribution of therapy‐related signatures in the five cases with available SMN samples. (A) The summary of the patients with available tumor samples. (B) The CNV analysis. (C) Relative contribution of therapy‐related signatures. ALL, acute lymphoblastic leukemia; AML, acute myeloid leukemia; EM, ependymoma; MB, medulloblastoma; PPB, pleuropulmonary blastoma; RCC, renal cell carcinoma; RMS, rhabdomyosarcoma; SBS; single base substitution; SBS‐MM1; mutational signature identified in multiple myeloma cases in the previous study (Supplementary methods).

We performed WES for the five cases for which SMN tumor specimens were available, including cases of AML (*n* = 2), ALL (*n* = 2), and renal cell carcinoma (*n* = 1) (Figure 3A). Two of the available specimens were a third cancer (AML in SMN03 and ALL in SMN04). Known somatic driver mutations in *PTPN11* (NM_002834: c.T211C: p.F71L) and *KRAS* (NM_004985: c.G35A: p.G12D) were detected in the ALL (SMN04) and AML (SMN08) samples.

### 3.4 Copy number alterations

The results of the copy number variant analysis are shown in Figure S1. In SMN03, in which a germline *TP53* pathogenic variant was identified, a deletion was detected in 17p13, including the remaining allele in the *TP53* region (Figure 3B and Figure S1).

### 3.5 TMB analysis

A high TMB (>10 mutations/Mb) was observed in two patients (SMN04 and SMN10) (Table S5), including one patient with a germline pathogenic variant of a mismatch repair (MMR) gene (*PMS2* in SMN04).

### 3.6 Mutational signature analysis

To evaluate the contribution of anticancer, agent‐induced DNA damage to SMN development, a mutational signature analysis was performed. A platinum drug‐related signature was observed in three cases (Figure 3C).

## 4 DISCUSSION

Our analysis revealed that germline genetic alterations contribute to SMN development following treatment of childhood solid cancers. First, we identified pathogenic variants in 35.7% of our SMN cohort. The prevalence was higher than that reported in a previous comprehensive study of primary cancer in pediatric patients (8.5%),^8^ and it was especially high in subsequent leukemia cases and cases with multiple episodes of SMN. The prevalence was also higher than that observed in a previous study of cancer survivors with SMN (6.4%).^13^ This discrepancy might result from differences in the SMN subtypes between cohorts due to different eligibility criteria. In the cancer survivor study cohort,^13^ most patients had survived longer than 10 years after diagnosis to at least 18 years of age, and few patients experienced subsequent leukemia. By contrast, our cohort was not limited to survivors, but included deceased patients. In fact, in our study, the median interval from primary cancer to SMN was 5.5 years, and more than 50% of the SMNs in our cohort were leukemia. Moreover, all five patients with pathogenic variants in CPGs subsequently developed leukemia, and three of them were deceased. These findings suggest a high prevalence of CPGs among patients with subsequent leukemia. Another study showed a high prevalence of pathogenic variants in CPGs in pediatric therapy‐related myeloid neoplasms (15%).^14^ The highest frequency of CPG variants in our cohort might be due to the selection bias that results from the retrospective collection of patients with second cancers and the inclusion of four cases with multiple SMNs.

Tumor genomic analysis revealed high TMB (>10 mutations/Mb) in an SMN specimen from SMN04 with a *PMS2* variant. Generally, compared with adult cancer, pediatric cancer is known for its low TMB.^20^ Moreover, the tumors of patients with a germline mutation in MMR genes, including *PMS2*, typically demonstrate high TMB.^21^ Interestingly, another case also had high TMB, although no pathogenic variant in CPGs was detected in the patient (SMN10). In this case, a MMR abnormality that cannot be detected by WES might be present in the germline. Evaluation of TMB in SMN samples may be contribute to the therapeutic strategy. In treating patients with high TMB, the use of immune checkpoint inhibitors has been suggested.^20^, ^22^

We found that three of five patients with a CPG variant had not received radiation therapy. Although radiation therapy is a risk factor for SMN development, avoiding such therapy is insufficient to reduce the risk of SMN in patients carrying CPG variants. Our analysis showed that a platinum‐induced mutational signature was common in SMN specimens from the patients treated with platinum during primary treatment, which is concordant with a previous study that reported that platinum‐based chemotherapy confers a risk of secondary leukemia development.^23^ The use of not only known high‐risk agents, such as topoisomerase II inhibitors, but also platinum agents might need to be minimized in treatment strategies for patients with a CPG variant.

## 5 CONCLUSION

Children with SMNs that developed after solid tumors had a high prevalence of pathogenic variants in cancer‐predisposing genes. We highlight that overlapping effects of genetic background and primary cancer treatment contribute to the development of second cancers after treatment of pediatric solid tumors. These results support universal germline genetic screening for children with cancer to assess the risk of SMN development.

## AUTHOR CONTRIBUTIONS

**Masanori Yoshida:** Conceptualization (equal); data curation (equal); formal analysis (equal); investigation (equal); methodology (equal); project administration (equal); resources (equal); software (equal); validation (equal); visualization (equal); writing – original draft (equal). **Kazuhiko Nakabayashi:** Data curation (equal); formal analysis (equal); investigation (equal); methodology (equal); resources (equal); software (equal); validation (equal); visualization (equal); writing – original draft (equal). **Wentao Yang:** Formal analysis (equal); methodology (equal); software (equal); visualization (equal); writing – original draft (supporting). **Aiko Sato‐Otsubo:** Formal analysis (equal); methodology (equal); software (equal); visualization (equal); writing – original draft (supporting). **Shin‐ichi Tsujimoto:** Resources (equal); validation (equal). **Hiroko Ogata‐Kawata:** Data curation (equal); formal analysis (equal); methodology (equal); software (equal); writing – original draft (supporting). **Tomoko Kawai:** Data curation (equal); methodology (equal); resources (equal); software (equal). **Keisuke Ishiwata:** Data curation (equal); methodology (equal). **Mika Sakamoto:** Methodology (equal); software (equal); supervision (supporting). **Kohji Okamura:** Methodology (equal); software (equal). **Kaoru Yoshida:** Resources (equal). **Ryota Shirai:** Resources (equal). **Tomoo Osumi:** Conceptualization (equal); resources (equal). **Chikako Kiyotani:** Resources (equal). **Yoko Shioda:** Resources (equal). **Keita Terashima:** Resources (equal). **Sae Ishimaru:** Resources (equal). **Yuki Yuza:** Resources (equal). **Masatoshi Takagi:** Resources (equal). **Yuki Arakawa:** Resources (equal). **Toshihiko Imamura:** Resources (equal). **Daisuke Hasegawa:** Resources (equal). **Akiko Inoue:** Resources (equal). **Takako Yoshioka:** Resources (equal). **Shuichi Ito:** Supervision (equal). **Daisuke Tomizawa:** Resources (equal); supervision (equal). **Katsuyoshi Koh:** Resources (equal); supervision (equal). **Kimikazu Matsumoto:** Resources (equal); supervision (equal). **Nobutaka Kiyokawa:** Resources (equal); supervision (equal). **Seishi Ogawa:** Methodology (equal); supervision (equal). **Atsushi Manabe:** Resources (equal); supervision (equal). **Akira Niwa:** Funding acquisition (equal); methodology (equal). **Kenichiro Hata:** Funding acquisition (equal); methodology (equal); software (equal); supervision (equal). **Jun J. Yang:** Conceptualization (equal); funding acquisition (equal); supervision (equal); writing – review and editing (equal). **Motohiro Kato:** Conceptualization (equal); funding acquisition (equal); investigation (equal); methodology (equal); project administration (equal); resources (equal); supervision (equal); writing – original draft (equal).

## CONFLICT OF INTEREST STATEMENT

The authors declare no potential conflicts of interest.

## ETHICAL APPROVAL STATEMENT

This study was approved by the Institutional Ethics Board of the National Center for Child Health and Development (#1025 and #1035), and required informed consent was obtained from the patients and/or guardians.

## Supporting information


Data S1

## Data Availability

The data that support the findings of this study are available from the corresponding author upon reasonable request.
