# Supplementary material for: Prevalence of pathogenic variants in cancer‐predisposing genes in second cancer after childhood solid cancers
Source: Cancer Med. 2023 Apr 6;12(10):11264–73. doi: 10.1002/cam4.5835 (PMC10242325; doi:10.1002/cam4.5835)
Supplement: Supplementary file 1 — Data S1 [file CAM4-12-11264-s001.docx]

**Supplementary Appendix**

Supplement to: Prevalence of pathogenic variants in cancer-predisposing genes in second cancer after childhood solid cancers

Supplementary Appendix 1

Supplementary methods 2

Sample preparation and whole-exome sequencing 2

Short read mapping and variant detection 2

Data analysis 2

Validation of detected germline variants 3

Mutational signature analysis 3

Supplementary Figure S1. The results of the CNV analysis 5

Supplementary Table S1. The specimens for whole-exome sequencing 6

Supplementary Table S2. Genes examined for germline variants 7

Supplementary Table S3. Pathogenic or likely pathogenic variants 8

Supplementary Table S4. Pathogenic or likely pathogenic germline mutations
in the control cohort 9

Supplementary Table S5. Tumor mutation burden in SMN samples 10

**Supplementary methods**

**Sample preparation and whole-exome sequencing**

To identify potential pathogenic variants in second malignant neoplasms, we performed whole-exome sequencing (WES). The peripheral blood or bone marrow samples during the tumor-free period or buccal swabs were collected as germline samples. (Table S1) DNA was extracted from bone marrow samples, peripheral blood, or buccal swab during complete remission using a QIAamp DNA Mini Kit (Qiagen, Hilden, Germany). After fragmentation of the DNA to approximately 200 bp with advanced focused acoustics (Covaris, Woburn, MA), library construction was performed with a combination of the SureSelect HumanAll Exon Kit (Agilent Technology, Santa Clara, CA) and the KAPA HyperPrep Kit (NIPPON Genetics, Tokyo, Japan) according to the manufacturers’ protocols. Then, enriched fragment libraries were sequenced on an HiSeq 2500 (Illumina, San Diego, CA) in 101-bp paired-end mode. Image analyses and base calling were performed using HiSeq Control Software version 2.2.68 and Real-Time Analysis Software version 1.18.66.3 (Illumina). Ball call files were converted to fastq files using bcl2fastq Conversion Software version 1.8.4 (Illumina).

**Short read mapping and variant detection**

Short reads obtained from the sequencer were processed, mapped, and analyzed according to a previous report with some modifications([1](#_ENREF_1)). Briefly, the paired-end reads were first trimmed by removing library adapters and low-quality bases at the ends and then aligned to the hs37d5 sequence (GRCh37 and decoy sequences) using the Burrows–Wheeler Aligner MEM. Uniquely mapped read pairs were selected to generate SAM and BAM files, followed by removal of PCR duplicates, local realignment, and recalibration of map quality scores. Multi-sample calling with diagnostic and remission samples using the Genome Analysis Toolkit (GATK) HaplotypeCaller was used to detect mutations. Annotations of altered sites were made using the ANNOVAR software based on GRCh37 with annotation databases, including SIFT ([2](#_ENREF_2)), Polyphen2 ([3](#_ENREF_3)), and CADD ([4](#_ENREF_4)).

**Data analysis**

First, to detect rare variants, variants with >0.1% population frequency in the 1000 Genomes Project were excluded. Moreover, the variants reported in both the 1000 Genomes Project (released August 2015) and the dbSNP (version 144) database were excluded. For quality control, variants that failed to pass the GATK filters were also eliminated. To extract the pathogenic variant, the truncation or missense variants in the coding region and missense variants in the splice region were included, while in-frame indels were excluded. Detected rare non-silent coding variants were assessed according to the guideline of the American College of Medical Genetics and Genomics and gene-specific online databases. Variant databases included NCBI ClinVar (http://www.ncbi.nlm.nih.gov/clinvar/), International Agency for Research on Cancer (http://p53.iarc.fr/), Breast Cancer Information Core (http://research.nhgri.nih.gov/bic/), Leiden Open Variation Database (http://chromium.lovd.nl/LOVD2/colon_cancer/home.php), and Catalogue of Somatic Mutations in Cancer (COSMIC, http://cancer.sanger.ac.uk/cancergenome/projects/cosmic).

**Validation of detected germline variants**

All detected pathogenic or likely pathogenic variants in the 162 genes were validated by Sanger sequencing. Primers were designed using Primer3. PCR was performed using the AmpliTaq Gold 360 Master Mix (Applied BioSystems, Waltham, MA) using the following parameters: 95 °C for 5 min, 95 °C for 30 s, 58 °C for 30 s, 72 °C for 40 s for 35 cycles, 72 °C for 5 min, and storage at 4 °C.

**Mutational signature analysis**

To extract mutational signatures, single nucleotide variants (SNVs) (hg19) were classified into 96 possible combinations according to their trinucleotide contexts and SNV classes (e.g., C to A, G, or T). All SNVs from 11 samples (6 with 6-Mercaptopurine treatment) were compiled into a 96 × 11 matrix. The matrix was fitted into known mutational signatures and identified by Bootstrap with a cosine similarity cutoff of 0.01 according to the method of Maura et al.([5](#_ENREF_5)). The contribution of each identified signature was measured as the relative amount (percent) per sample. Known mutational signatures were obtained from the COSMIC database (Mutational signatures V3, synapse.org ID: syn12009743) and from the publication by Li et al. (therapy-related signature A and B)([6](#_ENREF_6)).

**References**

1. Fukawatase Y, Toyoda M, Okamura K, Nakamura K, Nakabayashi K, Takada S*, et al.* Ataxia telangiectasia derived iPS cells show preserved x-ray sensitivity and decreased chromosomal instability. Scientific reports **2014**;4:5421 doi 10.1038/srep05421.

2. Kumar P, Henikoff S, Ng PC. Predicting the effects of coding non-synonymous variants on protein function using the SIFT algorithm. Nature protocols **2009**;4(7):1073-81 doi 10.1038/nprot.2009.86.

3. Adzhubei I, Jordan DM, Sunyaev SR. Predicting functional effect of human missense mutations using PolyPhen-2. Curr Protoc Hum Genet **2013**;Chapter 7:Unit7 20.

4. Kircher M, Witten DM, Jain P, O'Roak BJ, Cooper GM, Shendure J. A general framework for estimating the relative pathogenicity of human genetic variants. Nature genetics **2014**;46(3):310-5 doi 10.1038/ng.2892.

5. Maura F, Degasperi A, Nadeu F, Leongamornlert D, Davies H, Moore L*, et al.* A practical guide for mutational signature analysis in hematological malignancies. Nat Commun **2019**;10(1):2969.

6. Li B, Brady SW, Ma X, Shen S, Zhang Y, Li Y*, et al.* Therapy-induced mutations drive the genomic landscape of relapsed acute lymphoblastic leukemia. Blood **2020**;135(1):41-55.

**
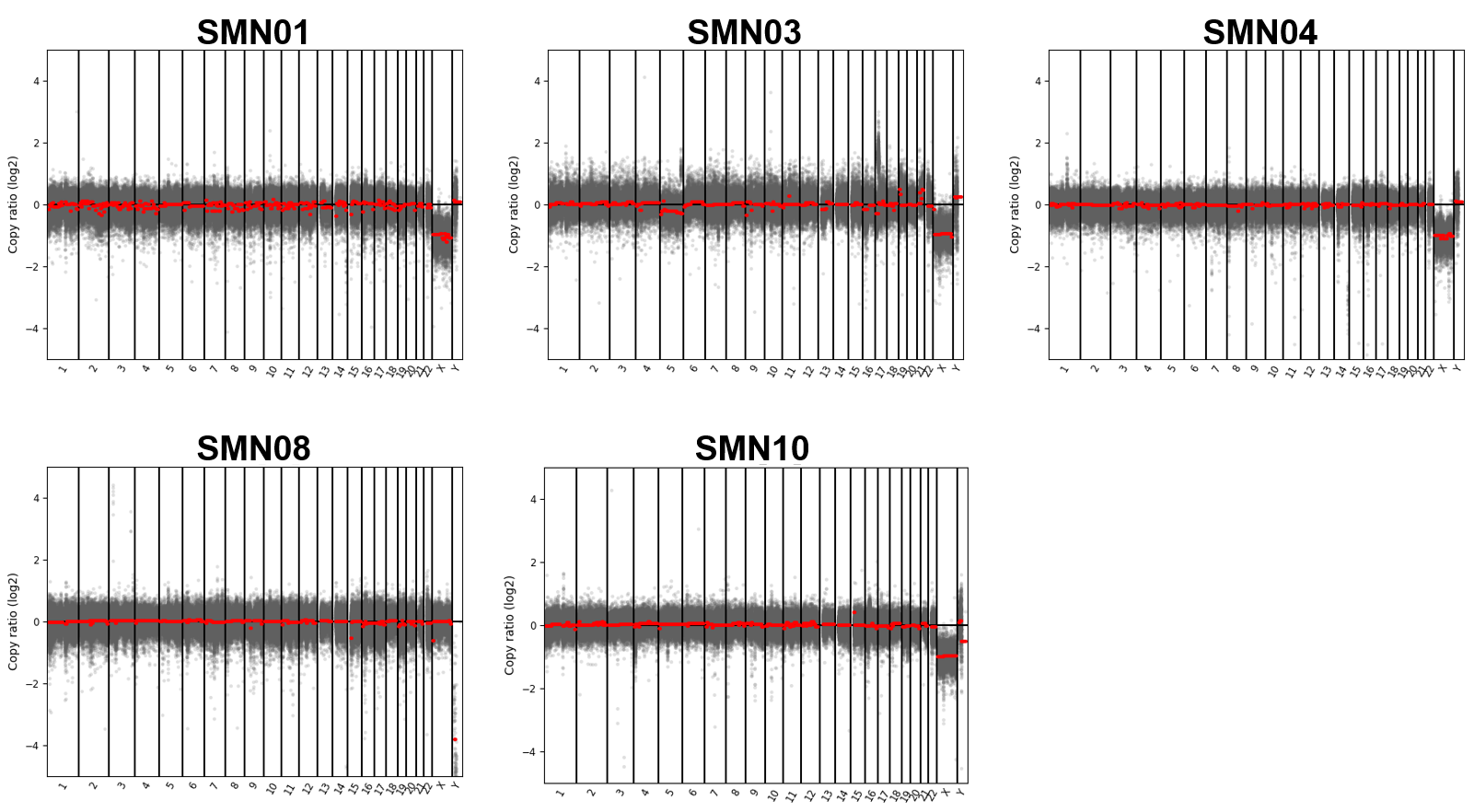
**

**Supplementary Figure S1. Results of the CNV analysis**

All chromosomes are shown on the X-axis, and the copy ratio (log2) is shown on the Y-axis.

**Supplementary Table S1. The specimens for whole-exome sequencing**

| **SMN No.** | **Primary cancer**  **subtypes** | **Second cancer**  **subtypes** | **Third cancer**  **subtypes** | **Germline sample** | **The timing of germline**  **sample collections** | **Somatic sample** |
| --- | --- | --- | --- | --- | --- | --- |
| 1 | PPB | BCP-ALL |  | PB | Primary cancer | BCP-ALL |
| 2 | ACC | AML |  | PB | Primary cancer |  |
| 3 | RMS | OS | AML | Buccal swab | Third cancer | AML |
| 4 | MB | DC | BCP-ALL | Buccal swab | Third cancer | BCP-ALL |
| 5 | MB | OS | AML | Buccal swab | Second cancer |  |
| 6 | NB | Thyroid cancer |  | PB | Second cancer |  |
| 7 | RMS | T-LBL | RCC | PB***** | Third cancer |  |
| 8 | RMS | AML |  | BM***** | Second cancer | AML |
| 9 | WT | Thyroid cancer |  | PB | Second cancer |  |
| 10 | Ependymoma | RCC |  | PB | Second cancer | RCC |
| 11 | RMS | AA |  | PB | Second cancer |  |
| 12 | ES | AML |  | PB***** | Second cancer |  |
| 13 | ES | Bladder cancer |  | PB | Second cancer |  |
| 14 | NB | SPN |  | PB | Second cancer |  |

PPB, pleuropulmonary blastoma; ACC, Adrenal cortex cancer; RMS, rhabdomyosarcoma; MB, medulloblastoma; NB, neuroblastoma; WT, Wilms tumor; ES, Ewing sarcoma; BCP-ALL, B-cell precursor acute lymphoblastic leukemia; AML, acute myeloid leukemia; OS, osteosarcoma; DC, duodenal cancer; T-LBL, T-lymphoblastic lymphoma; RCC, renal cell carcinoma; AA, anaplastic astrocytoma; SPN, solid pseudopapillary neoplasm; PB, peripheral blood; BM, bone marrow

*The germline samples from PB and BM were collected from morphologically tumor-free periods.

**Supplementary Table S2. Genes examined for germline variants**

| **Genes with dominant inheritance patterns** | | | | | | | | | | |
| --- | --- | --- | --- | --- | --- | --- | --- | --- | --- | --- |
| *ALK* | *CBP* | *CREBBP* | *FGFR3* | *HRAS* | *MITF* | *NSD1* | *PRSS1* | *RHBDF2* | *SMARCA4* | *TGFBR1* |
| *APC* | *CDC73* | *CYLD* | *FH* | *KCNQ1OT1* | *MLH1* | *NTRK1* | *PTCH1* | *RUNX1* | *SMARCB1* | *TMEM127* |
| *AXIN2* | *CDH1* | *DICER1* | *FLCN* | *KIT* | *MMR* | *PALB2* | *PTEN* | *SDHA* | *SMARCE1* | *TNFRSF6 (FAS)* |
| *BAP1* | *CDK4* | *EGFR* | *FMR1* | *KRAS* | *MSH2* | [*PAX5*](http://www.genenames.org/cgi-bin/gene_symbol_report?hgnc_id=8619) | *PTPN11* | *SDHAF2* | *SOS1* | *TP53* |
| *BMPR1A* | *CDKN1B* | *ELANE* | *GATA2* | *LZTR1* | *MSH6* | *PDGFRA* | *RAD51* | *SDHB* | *SRY* | *TSC1* |
| *BRAF* | *CDKN1C* | *EP300* | *GJB2* | [*MAP2K1*](http://www.genenames.org/cgi-bin/gene_symbol_report?hgnc_id=6840) | *MTAP* | *PHOX2B* | *RAD51C* | *SDHC* | *STAT3* | *TSC2* |
| *BRCA1* | *CDKN2A* | *EPCAM* | *GLI3* | [*MAP2K2*](http://www.genenames.org/cgi-bin/gene_symbol_report?hgnc_id=6842) | *NF1* | *PMS2* | *RAD51D* | *SDHD* | *STK11* | *UROD* |
| *BRCA2* | *CEBPA* | *EXT1* | *GNAS1* | *MAX* | *NF2* | *POLD1* | [*RAF1*](http://www.genenames.org/cgi-bin/gene_symbol_report?hgnc_id=9829) | *SETBP1* | *SUFU* | *VHL* |
| *BRIP1* | *CHEK2* | *EXT2* | *H19* | *MEN1* | *NFIX1* | *POLE* | *RB1* | [*SHOC2*](http://www.genenames.org/cgi-bin/gene_symbol_report?hgnc_id=15454) | *TERT* | *WT1* |
| *CBL* | *COL7A1* | *FGFR2* | *HMBS* | *MET* | [*NRAS*](http://www.genenames.org/cgi-bin/gene_symbol_report?hgnc_id=7989) | *PRKAR1A* | *RET* | *SMAD4* | *TFAP2A* | *XRCC3* |
| **Genes with recessive inheritance patterns** | | | | | | | | | | |
| *ABCB11* | *ERCC2* | *FANCG* | *MRE11A* | *SBDS* | *XPA* |  |  |  |  |  |
| *ATM* | *ERCC3* | [*FANCI*](http://www.genenames.org/cgi-bin/gene_symbol_report?hgnc_id=25568) | *MUTYH* | *SERPINA1* | *XPC* |  |  |  |  |  |
| *BLM* | *ERCC4* | *FANCL* | *NBN* | [*SH2B3*](http://www.genenames.org/cgi-bin/gene_symbol_report?hgnc_id=29605) |  |  |  |  |  |  |
| *BUB1B* | *ERCC5* | *FANCM* | *NBS1* | *SH2D1A* |  |  |  |  |  |  |
| *DDB2* | *FAH* | *FAND2* | [*NHP2*](http://www.genenames.org/cgi-bin/gene_symbol_report?hgnc_id=14377) | *SLC25A13* |  |  |  |  |  |  |
| *DHCR7* | *FANCA* | *GBA* | [*NOP10*](http://www.genenames.org/cgi-bin/gene_symbol_report?hgnc_id=14378) | *SLX4* |  |  |  |  |  |  |
| *DIS3L2* | *FANCC* | *GPC3* | *POLH* | *SMARCA2* |  |  |  |  |  |  |
| *DKC1* | [*FANCD2*](http://www.genenames.org/cgi-bin/gene_symbol_report?hgnc_id=3585) | *HFE* | *RECQL3* | *TRIM37* |  |  |  |  |  |  |
| *DOCK8* | *FANCE* | *ITK* | *RECQL4* | *WAS* |  |  |  |  |  |  |
| [*ERCC1*](http://www.genenames.org/cgi-bin/gene_symbol_report?hgnc_id=17072) | *FANCF* | *L2HGDH* | *RMRP* | *WRN* |  |  |  |  |  |  |

**Supplementary Table S3. Pathogenic or likely pathogenic variants**

| **SMN**  **No.** | **Gene** | **Category** | **Pathogenicity** | **Primary cancer**  **subtype** | **SMN**  **Subtype** | **Chr** | **Position** | **Ref** | **Alt** | **AA change** | **ClinVar** | **VAF** |
| --- | --- | --- | --- | --- | --- | --- | --- | --- | --- | --- | --- | --- |
| 01 | *DICER1* | AD | P | PPB | BCP-ALL | 14 | 95572501 | - | T | T955fs | NA | 58% |
| 02 | *TP53* | AD | P | ACC | AML | 17 | 7577539 | G | A | R116W | Pathogenic/  Likely pathogenic | 48% |
| 03 | *TP53* | AD | P | RMS | OS/AML | 17 | 7574018 | G | A | R205C | Pathogenic | 46% |
| 04 | *PMS2* | AD | P | MB | DC/BCP-ALL | 7 | 6027051 | G | A | Q449X | Pathogenic | 50% |
| 05 | *PTCH1* | AD | P | MB | OS/AML | 9 | 98239982 | G | - | L450fs | Pathogenic | 14% |

AD, autosomal dominant; P, pathogenic; PPB, pleuropulmonary blastoma; ACC, adrenal cortex cancer; RMS, rhabdomyosarcoma; MB, medulloblastoma; MDS, myelodysplastic syndrome; BCP-ALL, B-cell precursor acute lymphoblastic leukemia; AML, acute myeloid leukemia; OS, osteosarcoma; DC, duodenal cancer; AA, amino acid; VAF, variant allele frequency

**Supplementary Table S4. Pathogenic or likely pathogenic germline mutations in the control cohort**

| **No** | **Gene** | **Category** | **Pathogenicity** | **Chr** | **Start** | **End** | **Ref** | **Alt** | **AA change** | **ClinVar** |
| --- | --- | --- | --- | --- | --- | --- | --- | --- | --- | --- |
| Control 98 | *NTRK1* | AD | P | 1 | 156846219 | 156846219 | C | - | R548fs | Pathogenic |

AD, autosomal dominant, P, pathogenic, AA, amino acid

**Supplementary Table S5. Tumor mutation burden in SMN samples**

| SMN No. | Mutation counts | TMB (/Mb) |
| --- | --- | --- |
| 01 | 39 | 1.16 |
| 03 | 35 | 1.04 |
| 04 | 525 | 15.56 |
| 08 | 39 | 1.16 |
| 10 | 341 | 10.11 |
